# Silent Synapses, LTP, and the Indirect Parallel-Fibre Pathway: Computational Consequences of Optimal Cerebellar Noise-Processing

**DOI:** 10.1371/journal.pcbi.1000085

**Published:** 2008-05-23

**Authors:** John Porrill, Paul Dean

**Affiliations:** Department of Psychology, Sheffield University, Sheffield, United Kingdom; University College London, United Kingdom

## Abstract

Computational analysis of neural systems is at its most useful when it uncovers principles that provide a unified account of phenomena across multiple scales and levels of description. Here we analyse a widely used model of the cerebellar contribution to sensori-motor learning to demonstrate both that its response to intrinsic and sensor noise is optimal, and that the unexpected synaptic and behavioural consequences of this optimality can explain a wide range of experimental data. The response of the Marr-Albus adaptive-filter model of the cerebellar microcircuit to noise was examined in the context of vestibulo-ocular reflex calibration. We found that, when appropriately connected, an adaptive-filter model using the covariance learning rule to adjust the weights of synapses between parallel fibres and Purkinje cells learns weight values that are optimal given the relative amount of signal and noise carried by each parallel fibre. This optimality principle is consistent with data on the cerebellar role in smooth pursuit eye movements, and predicts that many synaptic weights must be very small, providing an explanation for the experimentally observed preponderance of silent synapses. Such a preponderance has in its turn two further consequences. First, an additional inhibitory pathway from parallel fibre to Purkinje cell is required if Purkinje cell activity is to be altered in either direction from a starting point of silent synapses. Second, cerebellar learning tasks must often proceed via LTP, rather than LTD as is widely assumed. Taken together, these considerations have profound behavioural consequences, including the optimal combination of sensori-motor information, and asymmetry and hysteresis of sensori-motor learning rates.

## Author Summary

The cerebellum or “little brain” is a fist-sized structure located towards the rear of the brain, containing as many neurons as the rest of the brain combined, whose functions include learning to perform skilled motor tasks accurately and automatically. It is wired up into repeating microcircuits, sometimes referred to as cerebellar chips, in which learning alters the strength of the synapses between the parallel fibres, which carry input information, and the large Purkinje cell neurons, which produce outputs contributing to skilled movements. The cerebellar chip has a striking resemblance to a mathematical structure called an adaptive filter used by control engineers, and we have used this analogy to analyse its information-processing properties. We show that it learns synaptic strengths that minimise the errors in performance caused by unavoidable noise in sensors and cerebellar circuitry. Optimality principles of this kind have proved to be powerful tools for understanding complex systems. Here we show that optimality explains neuronal-level features of cerebellar learning such as the mysterious preponderance of “silent” synapses between parallel fibres and Purkinje cells and behavioural-level features such as the dependence of rate of learning of a motor skill on learning history.

## Introduction

The uniformity of the cerebellar microcircuit [1] has long been attractive to modellers. The original Marr-Albus framework [2],[3] continues to be influential, particularly in the adaptive-filter form developed by Fujita [4] to deal with time-varying signals [5],[6]. However, although variants of the cerebellar adaptive-filter model are widely used and show great promise for generic motor control problems [7]–[13], they are typically constructed in a distributed form that makes mathematical analysis of their properties difficult. It is therefore still unclear whether the adaptive-filter model has the power and robustness needed to underlie the computational capacities of the cerebellum.

One method of addressing this question is to use a lumped version of the model, in simulated tasks that are simplified as much as possible while still retaining the computational demands of the real-world equivalent. This approach has indicated that, when wired in a recurrent architecture, the adaptive filter can use the *sensory* consequences of inaccurate movements for adaptive feedfoward control [14]–[17], thereby solving the classic problem of the unavailable motor-error signal [18],[19]. The recurrent architecture allows the filter to decorrelate an efference copy of motor commands from the sensory signal, ensuring that any remaining movement inaccuracies are not the result of the inadequate commands. The translation of ‘simple’ motor commands into the detailed instructions required for accurate movements has long been considered a central function of the cerebellum [2], and this translation entails the adaptive compensation of time-varying biological motor plant (muscles, tendons, linkages, etc.). The demonstration that the adaptive filter in a recurrent architecture can achieve adaptive compensation using only physically available signals is thus an important step towards establishing its computational suitability as a model of the cerebellar microcircuit.

A second requirement of a cerebellar model is robustness in the face of typically biological features of motor control problems. One ubiquitous example of such a feature is the presence of noise in biological signals [20]. In the modelling examples given above, both input and internal signals were assumed to be noise free. Here we investigate the performance of the model when noise is added to these signals. The investigation is in two parts. First, we show that an adaptive filter using the standard covariance learning rule behaves optimally with respect to input and internal noise. Secondly, we show there are important consequences of this computational optimality for both the neuronal implementation of the adaptive-filter, and for behavioural learning rates. These findings are significant for understanding not only cerebellar function, but also the relationship between computational and implementational aspects of neural modelling in general [21].

## Results

### Basic Model

**Figure 1:**
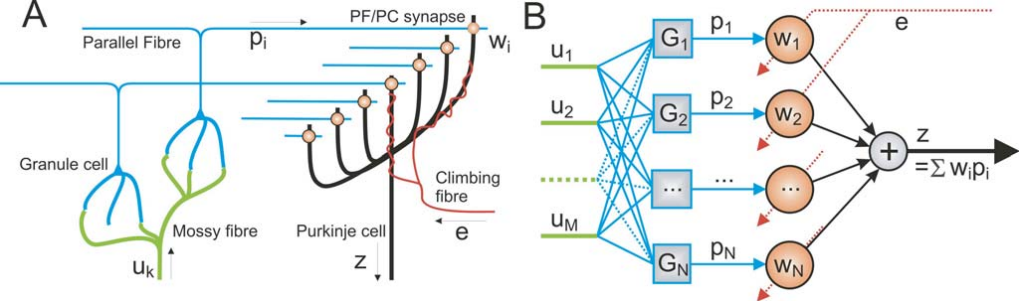
Schematic diagram of the organisation of the cerebellar microcircuit and its interpretation as an adaptable filter. (A) The mossy fibre input signals are distributed over many granule cells whose axons form parallel fibres (PFs) that synapse on Purkinje cells (PCs). In models of Marr-Albus type, correlated firing of a PF and the single climbing fibre (CF) which winds around the PC alters the efficacies of the PF/PC synapses. (B) Processing of MF inputs *u_k_*(*t*) by the granule cell layer is interpreted as analysis by a bank of causal filters *G_i_* so that the PFs carry signals which form an expansion re-coding *p_i_* = *G_i_*[*u*_1_,…,*u_M_*] of the MF inputs. PC output is modelled as a weighted sum *z*(*t*) = Σ*w_i_p_i_*(*t*) of its PF inputs so the PC implements a linear-in-weights filter *C* = Σ*w_i_G_i_*. The CF input is interpreted as a training signal *e*(*t*) which adapts synaptic weights *w_i_* using Equation 2; this hetero-synaptic covariance learning rule [23] is consistent with known properties of LTD and LTP at PF/PC synapses and is identical in form to the LMS learning rule of adaptive control theory.

The linear adaptive-filter model of the cerebellar microcircuit [4],[22] is outlined in Figure 1. Filter inputs correspond to mossy fibre signals, conveying information about the current sensory and motor state of the organism. These inputs are recoded by a bank of linear filters representing the granular layer, whose outputs (PF signals) are weighted (PF synapses on Purkinje cells) then summed to constitute the filter output (Purkinje cell firing). Weights are adjusted in response to an error signal (climbing fibre input to Purkinje cell), using the covariance learning rule [23]. This rule, which assumes that signals are carried by modulation of a tonic firing rate so that positive and negative values can be coded, is identical in form to the powerful Least Mean Square rule of adaptive control theory [24]. It requires bidirectional plasticity (that is both LTD and LTP) at synapses between parallel fibres (PFs) and Purkinje cells [25], so that synaptic weights decrease when climbing fibre input is positively correlated with parallel fibre input, and increase when the correlation is negative. If the filter is properly connected, this learning rule learns weights which combine parallel fibre inputs so that the PC output has minimal mean square error. It should be noted that in Figure 1 we follow the convention of referring only to parallel fibre synapses, without mentioning the synapses between the ascending axons of granule cells and Purkinje cells. However, the arguments in the paper would not be affected by inclusion of ascending axon synapses, provided their behaviour conformed to the covariance learning rule.

**Figure 2:**
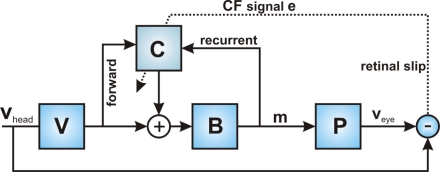
Architecture used for simulations of horizontal VOR adaptation. The task of the VOR is to convert the vestibular signal *v*_head_ into motor commands *m* to the oculomotor plant *P* which move the eye so as to exactly compensate head movements: *v*_eye_ = *v*_head_. We model this reflex as a fixed pathway through the brainstem supplemented by forward and recurrent adaptable pathways via the cerebellum. In previous work we have argued that VOR plant compensation depends mainly on the recurrent pathway through *C* [14],[15],[17], which has the advantage that the required teaching signal is sensory error, that is the retinal slip *e* as shown. Feedback-error learning [22] uses an alternative architecture without the recurrent loop; in this case, the required teaching signal is motor error, *e*_M_ = *P* ^−1^ *e*. In more general adaptation problems both pathways seem to be necessary, with one being well-adapted to vestibular compensation and one to plant compensation [17]. In all these architectures, the requirement for learning stability is that the teaching signal *e* must be related by a strictly positive real (SPR) transfer function to error in cerebellar output (which is trivially satisfied for the case of adaptation of scalar gain). Given the SPR assumption, our conclusions apply equally to all these configurations.

The uniformity of the cerebellar microcircuit implies that a model can be tested using any convenient cerebellar task. Adaptation of the vestibulo-ocular reflex (VOR) is relatively simple, has been extensively modelled and investigated [26], and previously used to investigate the computational properties of adaptive-filter models [14],[15],[17]. The simplified architecture used for the simulations is shown in Figure 2.

Horizontal VOR accuracy requires that motor commands to eye muscles compensate for changes in the dynamic properties of both the oculomotor plant *P* and of vestibular processing V. We have previously shown that plant compensation can be learnt by an adaptive filter version of the Marr-Albus algorithm using the recurrent pathway illustrated in Figure 2, in which the filter receives an efference copy of the motor commands to the plant. In contrast, the forward pathway shown in Figure 2 is suitable for compensating for changes in vestibular processing. In the simulations below both architectures are used although, since these simulations deal only with changes in scalar gain, this is not a crucial distinction.

### Learning Rule Optimises Filter Weights for Noisy Signals

In general, appropriately connected adaptive-filters using the covariance learning-rule will achieve optimal filter weights that minimise the error measure *e* (Figures 1 and 2). Since *e* is a measure of task performance, these weights enable the filter to perform the task accurately. This optimal behaviour clearly generalises to the situation where noise is present in PF signals: because this noise affects the filter output, minimising *e* will also tend to minimise the effect of PF noise, by choosing weights that are optimal for eliminating disturbances due to PF noise.

**Figure 3:**
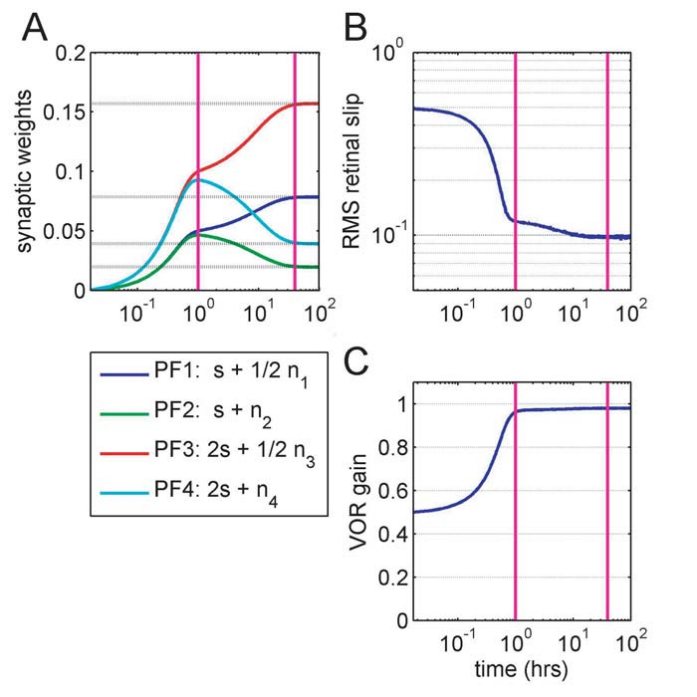
Synaptic weights are optimal with respect to output noise. Here the plant suffers a 50% decrease in gain and learning takes place in 4 PFs carrying levels *α*_i_ = 1, 1, 2, and 2 of the same unit power signal *s* and levels *σ*_i_ = 1/2, 1, 1/2, and 1 of independent noise (see legend below for [A]). During the fast learning phase, synaptic weights are learned that are approximately proportional to signal amplitude *α*_i_ on the relevant PF ([A]—the learning rate was chosen to make this phase last ∼1 h, as shown by the first vertical line). During this phase, performance improves dramatically (B) and overall VOR gain approaches a value just smaller than unity (C). This is followed by a slow learning phase (predicted length shown by second vertical line) in which weights rearrange themselves to be proportional to *α*_i_/*σ*_i_^2^ (predicted values shown by dotted lines). During this stage there is a small improvement in performance as the effect of the disturbance is minimised, but overall VOR gain is virtually unaffected.

The optimality principle can be illustrated by considering the case where a number of PFs carry signals *p_i_* with different levels α*_i_* of a signal of interest *s* but contaminated by independent noise components *n_i_* of power σ_i_^2^. It is shown in the Methods section (Equation 6) that mean square output error is minimised when the weights on these input signals have the ratios *w_i_∶w_j_* = α*_i_*/σ_i_^2^∶α*_j_*/σ_j_^2^. Figure 3 shows the time course of this learning, for the case where plant gain is suddenly decreased from 1.0 to 0.5. and the filter has four PF channels carrying differing amounts of efferent-copy signal and noise.

Figure 3A illustrates a general phenomenon for low levels of PF noise, namely the existence of fast and slow phases of learning (see Methods). Initial learning is fast, producing a 5-fold drop in retinal slip error in ∼60 batches, nominally about 1 hour of input. During this phase the weight vector converges close to the subspace of weight combinations which performs the task in the absence of noise. Thus, the values of the weights attained after this early fast phase of learning are sufficient to achieve a near optimal VOR gain of just below 1.0. Subsequent learning is much slower (note log scale for *x*-axis), as the weight vector moves essentially within this subspace to bring all weights to the optimal values determined by Equation 6. During this learning phase performance improves, but less dramatically, as the smaller noise contribution to task errors is reduced. The slowest time constant for this phase of learning is lengthened by a factor approximately equal to the signal to noise ratio (Equation 10).

### Implications for Sensory Processing

If the signals carried by parallel fibres correspond to a set of noisy sensory estimates of an environmental property, and appropriate cost information is carried on the climbing fibre, the adaptive-filter behaviour above leads to the optimal linear estimator in the Bayesian sense. Our analysis shows this explicitly for the simplest case of a minimum least square error estimator when the sensory estimate noises are independent. Such statistically optimal performance has been observed for humans integrating visual and haptic information [27], and the above result suggests that the adaptive-filter model of the cerebellum can match the performance of the whole subject. This result has particular relevance to smooth pursuit, a class of eye-movement known to be dependent upon the cerebellum, whose accuracy (in the initial open-loop phase) appears to be limited primarily by sensory noise [28]. This example is considered further in the Discussion.

### Implications for Weight Values

**Figure 4 pcbi-1000085-g004:**
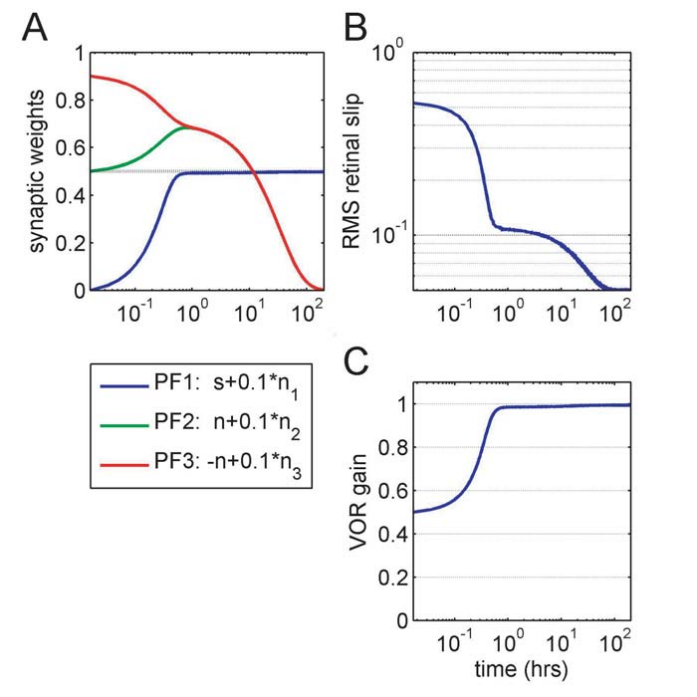
Covariance rule eventually drives weights on nuisance inputs to zero. In this simulation, the cerebellar input is carried on three parallel fibres. One (PF1) carries the required motor command *s*, and the other two (PF2, PF3) carry equal and opposite versions of a nuisance signal *n* with the same power as, but uncorrelated with, the motor command. In addition, each parallel fibre carries a small additive component of noise *n_i_* with (σ = 0.1) representing intrinsic PF noise which is uncorrelated between parallel fibres (see legend below [A]). The initial synaptic weights on PF1, 2, and 3 are set to 0, 0.5, and 0.9, respectively. (A) shows that on a fast time scale, the signal synaptic weight converges to a value where the plant is compensated, and over the same time scale, the nuisance signal weights converge to equal values so that the correlated nuisance signal they carry cancels. On this time scale, performance improves dramatically. The non-zero weight values on the nuisance inputs are not stable, however, and the small component of intrinsic noise drives them to zero on a slower time scale. This process is associated with a smaller improvement in performance (shown in [B] and [C]).

It can be seen from Figures 3 and 4 that even weights for parallel fibres carrying relevant signals are driven to low values if they also carry high amounts of additive noise. We now consider a second type of noise, namely potentially useful signals carried by PFs but which are irrelevant to the current task (termed ‘nuisance’ signals in the control-theory literature), these signals could be correlated between different PFs. A simple example would be a parallel fibre that carries information about the conditioned stimulus in classical conditioning. Conditioned stimuli are deliberately chosen on the basis of their not having prior influence on the response to be conditioned, so before acquisition commences the corresponding parallel-fibre signal is essentially all noise. Its weight will therefore have been set to zero at the fast time scale before the start of formal training.

A more interesting example is provided by the case of two parallel fibres, one carrying irrelevant information *n* and a second fibre carrying the same information with the opposite sign *−n*. Here the total contribution to the task will be zero if the weights are equal. From any arbitrary non-zero starting weights this state will be reached on the fast time scale. However if these PFs also carry an independent second component of noise (as they surely will) these redundant weights will go on changing to become zero on the slow time scale (illustrated in Figure 4). In general all non-zero weight combinations for which nuisance sources cancel will be unstable due to intrinsic noise. In a similar way large numbers of nuisance sources might cancel to good accuracy due to the central limit theorem, but their weights will nevertheless eventually converge to zero due to intrinsic noise.

### Implications for Neuronal Implementation

Parallel fibres are thought to carry a widespread array of information about the sensorimotor context in which motor activity takes place, including sensory signals, copies of motor commands, and signals about the state of the organism such as arousal [29]. The fact that there are so many (∼170,000) parallel-fibre inputs to a given Purkinje cell [6] implies that most parallel fibres will inevitably carry information which is only weakly related (low signal to noise) or is simply unrelated (all noise) to a given task. From the analysis above the long-term optimal synaptic weights for such synapses will be small or zero. Hence it is a consequence of the optimal performance of the model that most synapses between parallel fibres and Purkinje cells are expected to be silent, consistent with experimental evidence [30]–[32].

The second consequence of optimal performance is related to the first. In simplified computational models it is often assumed that a given synaptic weight can be either positive or negative. The fact that actual synapses do not change between excitatory or inhibitory forms can be finessed if the weights vary around some intermediate positive (or negative) value. However, if many of them are typically zero at the start of learning, the model can only be properly implemented if there is a second pathway from granule cells to Purkinje cells of opposite sign to the first, else learning would only be possible when it required Purkinje cell excitability to *increase*. Fortunately, this requirement appears to be consistent with recent experimental evidence indicating that there is climbing-fibre controlled plasticity in the synapses between parallel fibres and stellate and basket cells, which are inhibitory interneurons that project to Purkinje cells [31],[32]. Thus there is a second, indirect, pathway from granule cells to Purkinje cells via inhibitory interneurons that can support the learning required by the adaptive-filter model.

The final consequence is almost a triviality. Clearly if most synapses are silent they are not available for long-term depression (LTD). Hence for a large class of tasks learning must initially proceed via long-term potentiation (LTP), in either the direct or indirect pathway from granule cells to Purkinje cells. LTP in the direct excitatory pathway would increase Purkinje cell excitability, whereas LTP in the indirect inhibitory pathway would reduce Purkinje cell excitability. The covariance learning rule thus implies that LTP and LTD are in general of equal significance, rather than cerebellar LTP merely playing a book-keeping role by normalising an LTD-lead learning process. The predominance of silent granule synapses goes further by implying that LTP may be particularly important for new learning.

### Implications for Learning

The basic simplicity of the Marr-Albus mechanism as exemplified by the adaptive-filter model is substantially modified by the implementation issues just considered, in particular by the presence of both direct excitatory and indirect inhibitory pathways from granule cells to Purkinje cells. We have shown that synaptic positivity requires an indirect pathway whenever a task requires synaptic weights to be negative. Hence the locus of synaptic plasticity, in the direct or indirect pathway, will depend on the direction of the change to be learnt. This means that any differences between direct and indirect pathways will lead to asymmetries in learning behaviour.

**Figure 5 pcbi-1000085-g005:**
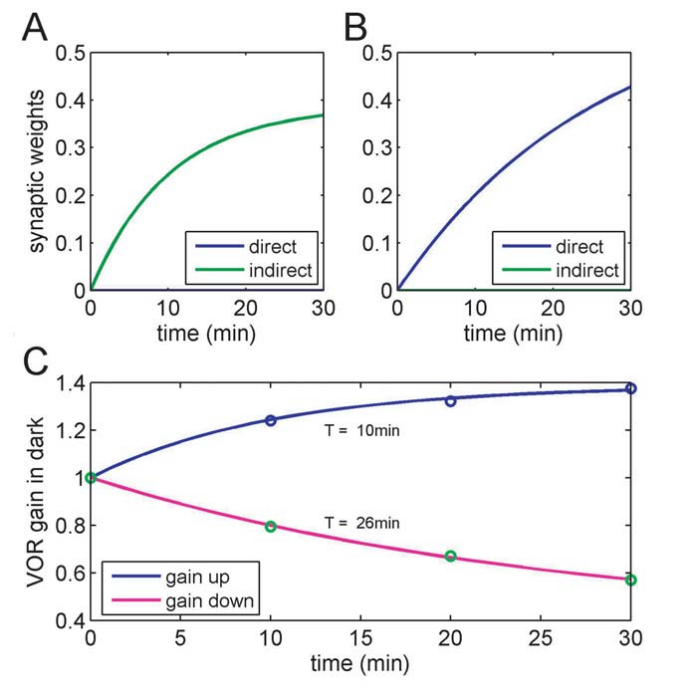
Effect of asymmetry between direct excitatory and indirect inhibitory pathways. Vestibular compensation experiment simulated in forward architecture. Signs for the vestibular signal were chosen so that the direct excitatory pathway learned gain down and the indirect inhibitory pathway learned gain up ([A] and [B], respectively). The asymmetry between the two pathways was chosen to be a difference in learning rates. (C) shows that data (circles) from Figure 1E of Boyden and Raymond [33] were well-fitted when the learning rate in the indirect pathway was about 9 times faster than that in the direct pathway, leading to time constants of 10 min for gain up and 26 min for gain down. A similar result (not shown) was obtained assuming a factor of 3 asymmetry between indirect and direct signal strengths.

An example is given in Figure 5, which illustrates the behaviour of a system with vestibular inputs arriving on both the direct excitatory pathway and an indirect inhibitory pathway. Signs were chosen so that gain down would initially be learnt by LTP on the direct pathway, consistent with [33]. It is further assumed that the learning rate in the direct pathway is smaller than that in the indirect pathway. The effect of these assumptions is to produce asymmetrical learning rates, with gain-up learning being about twice as fast as gain down (a similar result can be obtained using equal learning rates but with the indirect pathway carrying a more powerful signal than the direct pathway). This difference is similar to that found for VOR adaptation in the mouse [33]. Figure 5 therefore shows how, in principle, the presence of a direct excitatory and indirect inhibitory pathway could contribute to an observed asymmetry in learning rate. Additional differences between these pathways with respect to, for example, generalisation could also contribute to other kinds of experimentally-observed learning asymmetries (see Discussion).

**Figure 6:**
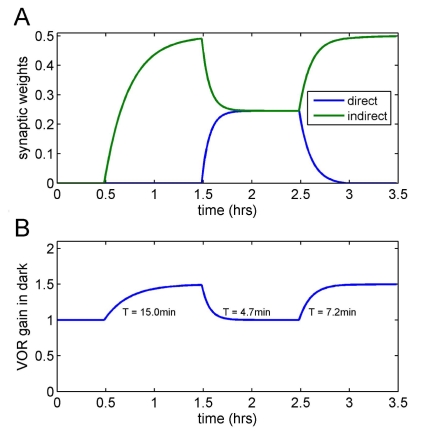
Hysteresis in vestibular compensation simulation in forward architecture. In response to a decrease in plant gain (top plot), learning proceeds initially via LTP in the direct pathway (A) and increases VOR gain in the dark by 50% (B); the learning rate has been adjusted to give this stage a time constant of 15 min. When the plant gain is returned to its original value, learning occurs in both pathways: via LTD in the newly available synapse in the indirect inhibitory pathway, and via LTP in the synapse in the direct excitatory pathway, leading to faster learning (with a time constant of approximately 5 min) during this gain down phase of learning. This is followed by a second phase of gain up learning, again increasing VOR gain by 50%. Learning is still possible at both sites, and this gain up phase has a faster time constant (approximately 7 min) than the initial gain up phase.

Finally, we have argued above that, if most synapses are inactive, learning novel tasks must proceed mainly via LTP. However once learning has taken place, these newly active synapses become available for learning via LTD. Hence the number of active synapses and the magnitude of the synaptic weight available for LTD will depend on previous experience, ensuring that learning rates will depend on previous learning history. An example of this hysteresis mechanism is given in Figure 6, which illustrates learning rates for an increase in VOR gain in the dark from 1.0 to 1.5, followed firstly by a decrease back to 1.0, then by another gain increase to 1.5. It is assumed that all weights are zero at the start of learning, and that the direct excitatory and indirect inhibitory pathways have identical signal strengths and learning rates. It can be seen that the initial learning of the gain increase (‘acquisition’) is slower than learning the subsequent decrease (‘extinction’), and also slower than re-learning the gain increase (‘re-acquisition’). As with the previous figure, Figure 6 shows how in principle the presence of direct and indirect pathways could contribute to hysteresis in learning rates.

## Discussion

An important step in evaluating candidate models of the cerebellar microcircuit is to assess their computational power. We show here that the adaptive-filter version of the Marr-Albus framework using the covariance learning rule has the very desirable computational property of providing optimal estimates of sensory input signals from the information available in the parallel fibres. This is important both for the general reason that noise is ubiquitous in neural signals, and more specifically because there is evidence suggesting that the cerebellum itself can make optimal use of noisy signals.

### Optimal Cerebellar Performance in Smooth Pursuit

Analysis of inaccuracies in open-loop smooth pursuit movements indicates that more than 90% of the variance arises from errors in sensory estimation of the speed, timing and direction of target motion [28], and that pursuit thresholds are similar to perceptual thresholds [34]. Since smooth pursuit is dependent upon the cerebellum (e.g., [35]), these findings suggest that the cerebellum can process noisy sensory information as well as the perceptual system as a whole. Moreover, at least in some instances perceptual processing of this kind has been shown to be statistically optimal (e.g., [27]). Recordings from smooth-pursuit related Purkinje cells in the cerebellar floccular complex suggest that variability in their open-loop responses is also driven primarily by sensory noise, with noise downstream from the Purkinje cells being of minor importance [36]. These findings together suggest that smooth pursuit performance is close to optimal given the noise present in sensory measurements, and that the cerebellum can make optimal use of those measurements. An important criterion, therefore, for assessing cerebellar models is their computational ability to reproduce such optimality.

### Complexity of Neuronal Implementation

A second feature of the present findings is the implication of the model's computational power for its implementation and performance. After long periods of training most of the model's weights are likely to be small or zero, consistent with recent experimental evidence [30]–[32]. We comment on four features of this finding.

1. The presence of many silent synapses may appear puzzling, given that *in vitro* studies of LTD typically report reductions in efficacy of only ∼50%. However, from the computational perspective the crucial point is whether the synapses are *functionally* silent, i.e. they do not influence Purkinje cell output. In fact Isope and Barbour [30] found that “… a large fraction of these synapses is so weak as to produce no detectable response” (p. 9676), and evidence from *in vivo* studies suggests that LTD is able to render parallel-fibre synapses on Purkinje cells functionally silent [31],[32]. The relationship between *in vitro* and *in vivo* LTD is an intriguing issue, but not directly germane to the central purpose of the present study.
2. As explained in the Results section, the presence of many silent synapses implies the necessity for a second pathway from granule cell to Purkinje cell, of opposite sign to the first and also capable of plasticity in accordance with the covariance learning rule. Again, recent experimental evidence is consistent with this requirement [31],[32]. This evidence also shows that the synapses between parallel fibres and interneurons in this pathway too are mainly silent, as our computational analysis would predict.
3. Perhaps unexpectedly, the addition of an indirect inhibitory pathway to the model's implementation substantially increase the complexity of its behaviour. Unless the direct and indirect pathways have identical properties, then learning tasks that engage them to different degrees will show differences in such properties as rate of learning (Figure 5), and exact nature of what is learned, as revealed for example by generalisation tests. Such differences have been demonstrated for gain-up and gain-down VOR learning [26], [33], [37]–[39], and our results show that a candidate explanation for the asymmetric learning rates is (a) the two tasks engage the direct excitatory and indirect inhibitory pathways to differing extents, and (b) the two pathways have different learning rates.
4. Finally, even if the two pathways were to have identical properties, a ‘new’ task (starting from zero weights in both pathways) will show learning rate hysteresis (Figure 6) as has been observed for VOR adaptation [33],[40],[41] and classical eyeblink conditioning [42],[43]. These observations establish that the presence of direct and indirect learning pathways is likely to contribute to learning-rate asymmetries and hysteresis, but of course do not rule out possible contributions from other sources, such as sites of plasticity in brainstem for the VOR or forebrain for classical conditioning, or in the granular layer for cerebellar learning in general.

### Interpretation of Behavioural Experiments

The possibility that cerebellar learning can proceed via at least 4 separate processes (LTP and LTD in either pathway) complicates the interpretation of behavioural studies in which one or more of those processes are compromised. As can be seen from Figures 5 and 6, the contribution of each process depends both on the direction of learning, and the organism's past history. For example, the neural bases of a new learning task (possibly the initial acquisition of eyeblink conditioning to a tone) may differ from those of an ongoing familiar task (VOR or saccadic calibration). This complication may contribute to the difficulty of identifying these neural bases using behavioural studies of mutants [44], though again it must be emphasised that there are a number of other possible sources contributing to difficulty in this area.

A related issue concerns the processes underlying the fast and slow phases of learning illustrated in Figures 3 and 4. It can be seen that in principle there could be some tasks where early learning uses a single process, whereas later learning uses a mixture (e.g., Figure 3A). Although a distinction between fast early learning (‘acute’) and slow subsequent learning (‘chronic’) is familiar in the cerebellar literature [26],[44], the mechanisms illustrated in Figure 3 have not so far been considered as a possible basis. One additional implication of this figure is that the slow acquisition of many motor skills (to expert level) might be caused in part by cerebellar input noise.

### Comparison With Other Cerebellar Models

The cerebellar algorithm we have described necessarily inherits the well-known optimality properties of the adaptive filter [24]. We have demonstrated statistical optimality explicitly and examined its consequences for a class of noisy inputs likely to be of importance in cerebellar learning. As far as we are aware, the cerebellar model described here is at present the only one demonstrated to guarantee statistical optimality in dealing with noisy inputs, and thus the only one known to be capable of, for example, the optimal smooth pursuit performance described experimentally [28],[34],[36].

There is an alternative account, however, of the experimentally observed preponderance of silent synapses between parallel fibres and either Purkinje cells or interneurons. The relation between weight distribution and storage capacity has been examined for perceptron models [45], and the optimal distribution has been shown to contain a high proportion of very weak or silent synapses. This analysis is based on the assumptions i) that the cerebellar microcircuit acts like a perceptron in which both inputs and outputs are binary and ii) that weights are distributed so as to achieve maximum storage capacity. Under these assumptions it is shown that coding capacity is maximised when 50% of weights are silent, and that this proportion increases if a noise threshold is introduced to increase reliability of classification.

Although the derivation is rigorous, there is a question of how far the Perceptron is in fact a suitable representation of the cerebellar microcircuit in a motor control context. Although Perceptrons have been used as models for cerebellar cortex based on the Marr-Albus framework [46],[47] they are not usually applied to motor control problems where continuous time-varying signals are required. In general the adaptive filter interpretation is more suited to these sensori-motor applications, and it is more closely linked to theoretical developments in adaptive control. Moreover, the task of learning the coefficients of an adaptive filter is very unlike that of coding many random bit patterns with a single template. For example in a motor control problem the inputs would generally be confined to a low dimensional subset of input space, an assumption that is basic to current machine learning algorithms such as locally weighted linear regression [48]. In these circumstances the requirement of maximising coding capacity is not relevant.

### Levels of Analysis

Although the simplicity of the Marr-Albus algorithm may seem to imply correspondingly simple learning behaviour, we have shown how constraints at the hardware level can mask this algorithmic simplicity so that Marr-Albus systems exhibit complex phenomena such as multiple time scales, asymmetry and hysteresis. Marr [21] distinguished between the computational, algorithmic and hardware levels of description in models of neural information processing. In fact models often have the greatest explanatory power when they integrate information across all three levels. Our previous work has concentrated primarily on the interaction between the two higher levels [14]–[17]. Here we have extended this work to include two important hardware level constraints, namely system noise and weight positivity, and show that they have computational consequences which are critical to understanding neuronal and behavioural aspects of cerebellar learning. It is of interest that recent experimental work on VOR adaptation has emphasised the complexity of the learning processes involved [26]. The results here suggest that such complexity is not in principle incompatible with the original Marr-Albus framework.

## Methods

### Simulations

In the simulations the model architecture shown in Figure 2 was programmed in MATLAB with *V*, *P*, and *B* taken as scalar gains. In recurrent architecture *V* was a unit gain and the forward pathway through *C* was not used giving an overall loop gain of *BP*/(1−*BC*). Initially *P* = 1, *B* = 1 so the plant is initially perfectly compensated when *C* = 0. For example when *P* is reduced to 0.5 exact compensation requires *C* = *B* ^−1^−*P* = 0.5. In adaptive filter models the cerebellar filter *C* analyses its input *m(t)* into many parallel fiber signals *p_i_* which are re-synthesized to form the output *z* = Σ *w_i_p_i_*. Since the simulations here deal only with scalar gains we do not require *p_i_* containing information about the past history of *m* as in our previous work. Assumptions about the nature of the *p_i_* are described separately for each simulation. Since the time dependence of the inputs is irrelevant to learning a scalar gain the input was taken to be constant. All noise signals were represented as white noise, results would be the same for other types of noise with the same variance and correlations.

The learning rule (Equation 2 below) at the parallel fibre/Purkinje cell synapse was implemented as a batch update rule, accumulating the total change in weight over the batch for fixed weights and then updating at the end of the batch. A batch consisted of 6,000 time steps so that with *dt* = 0.01 s a batch had a nominal duration of 1 min.The teaching signal *e* was retinal slip *v*_head_-*v*_eye_. The learning rate β was chosen to fix the fast time scale for each simulation. Although batch update was used for efficiency the results are essentially identical for continuous time update. The code for the all the simulations is available in Dataset S1.

### Analysis

The mossy fibre inputs to the granule cell layer are expansion-recoded as parallel fibre signals *p_i_* (note that these signals are assumed to be carried by modulation of a tonic firing rate so that both positive and negative signal values can be coded). These parallel fibre inputs are re-combined by the Purkinje cell to produce its output
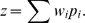
(1)If the desired output is γ*s* (i.e., the required gain is *γ*), the error in PC output is *z*−*γ* *s*. Learning stability requires that the climbing fibre input *e* is an approximation to this output error; that is, *e*≈*z*−*γ* *s*. The level of approximation required is that these quantities be related by a strictly positive real transfer function [49]. It has been shown that in recurrent architecture *e* can be an error in task space, that is, a sensory error, while forward architectures such as feedback-error learning require that *e* be a motor error signal. This distinction (discussed further in [15],[16]) is not relevant to the phenomena discussed here. The learning rule is the covariance learning rule
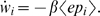
(2)If the strict positive realness condition is satisfied this learning rule can be shown to minimise mean square error *E* = 〈*e* ^2^〉.

We consider an illustrative situation in which each parallel fibre carries a combination of the signal of interest *s* and uncorrelated noise *n_i_*

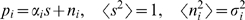
(3)(the noise sources are assumed to be pairwise uncorrelated). The mean square error has the form
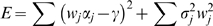
(4)whose minimum is at
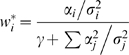
(5)so that the optimal weights are in the ratios
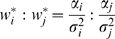
(6)Note that in general the optimal weights give an optimal gain 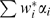, which is smaller than γ, this is due to the usual trade-off between bias and variance for an optimal estimator.

The rate of approach to the optimal weights is determined by the covariance learning rule which takes the form
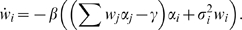
(7)Rigorous bounds on the time constants of this system can be obtained using the eigenvalue interlacing theorem [50], here we use a simpler heuristic approach. Suppose there was zero noise. Then weight update would take place entirely in the direction (α*_i_*) with time constant
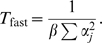
(8)


Superimposed on this is a motion in each coordinate direction generated by the noise term with time constants
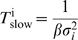
(9)(given subscripts fast and slow because noise power will usually be much smaller than signal power). The ratio of the slow to fast time constants is thus determined by the signal to noise ratio:
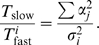
(10)


## Supporting Information

Dataset S1 Zipped folder containg MatLab code to generate Figures 3–6.
(1.23 MB ZIP)
