# Correction: Silent Synapses, LTP, and the Indirect Parallel-Fibre Pathway: Computational Consequences of Optimal Cerebellar Noise-Processing

**DOI:** 10.1371/annotation/349e7f3a-c9ce-4de2-b369-254ae8ab76cd

**Published:** 2009-01-24

**Authors:** John Porrill, Paul Dean

An incorrect version of Dataset S1 was presented. The full Dataset S1 file can be found here:
