# Supplementary figures and images for: Correction: Silent Synapses, LTP, and the Indirect Parallel-Fibre Pathway: Computational Consequences of Optimal Cerebellar Noise-Processing
Source: PLoS Comput Biol. 2009 Jan 24;5(1):10.1371/annotation/349e7f3a-c9ce-4de2-b369-254ae8ab76cd. doi: 10.1371/annotation/349e7f3a-c9ce-4de2-b369-254ae8ab76cd (PMC2641020; doi:10.1371/annotation/349e7f3a-c9ce-4de2-b369-254ae8ab76cd)

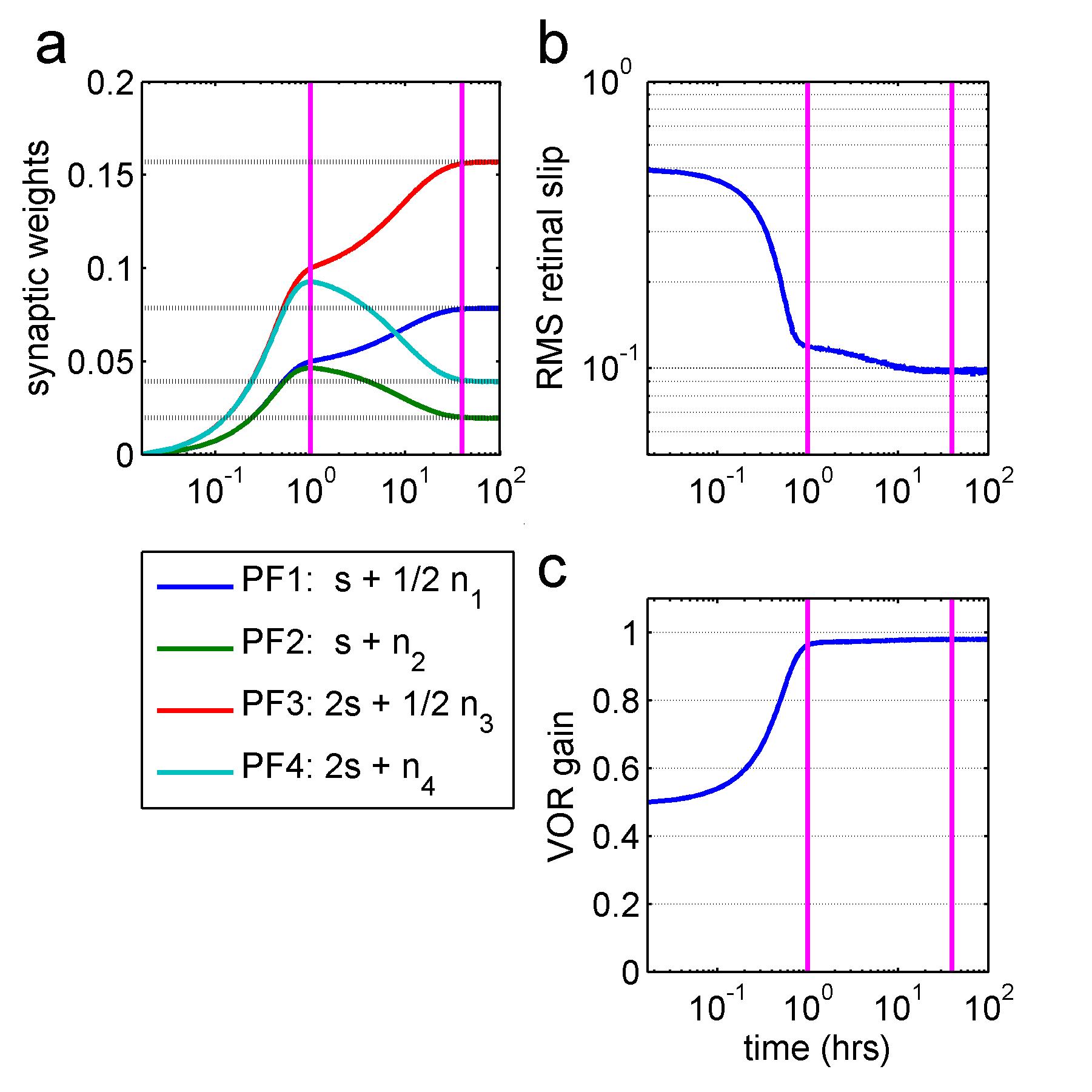

Supplement: Supplementary file 1 [file pcbi.349e7f3a-c9ce-4de2-b369-254ae8ab76cd.s001.zip › code/fig3/fig3.jpg]

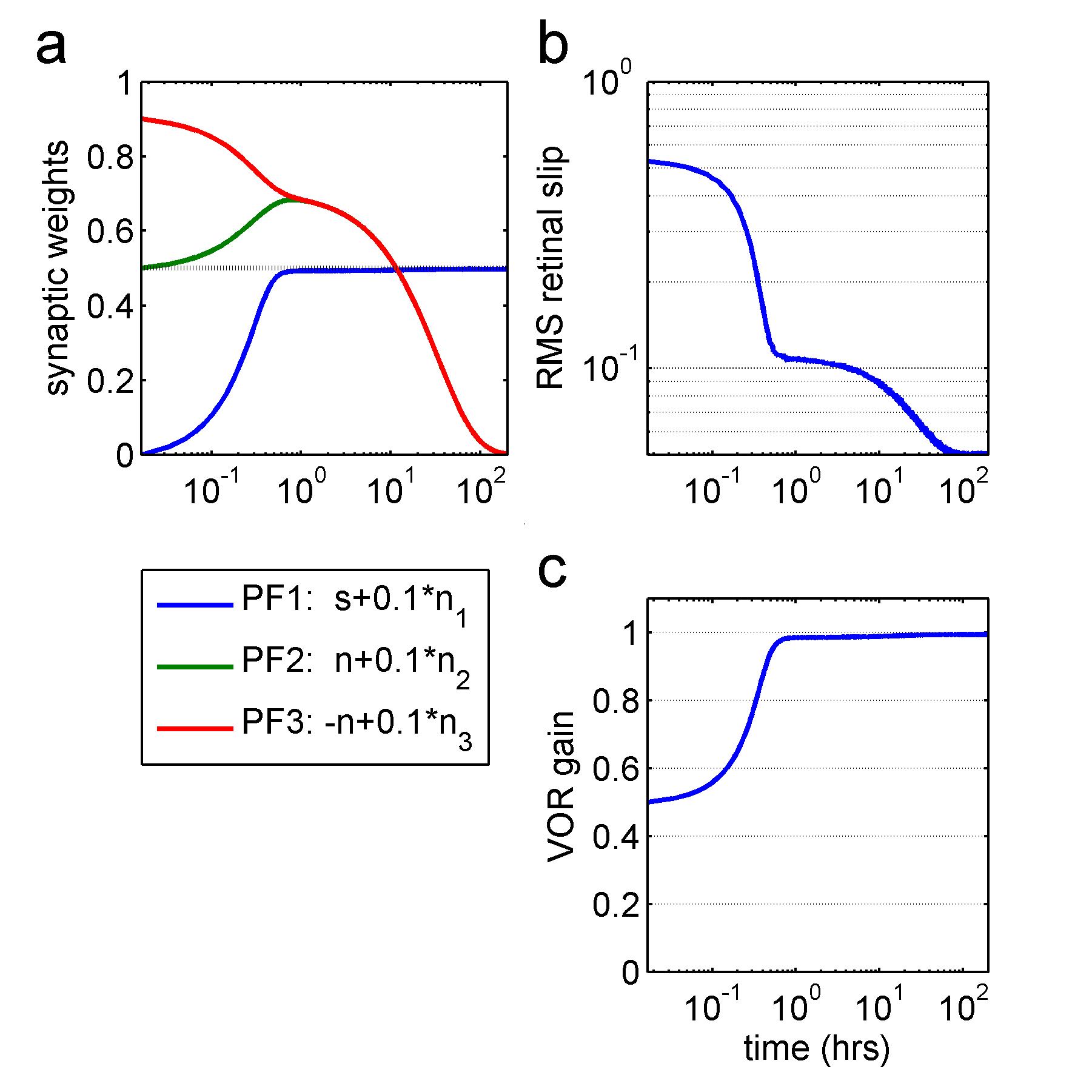

Supplement: Supplementary file 1 [file pcbi.349e7f3a-c9ce-4de2-b369-254ae8ab76cd.s001.zip › code/fig4/fig4.jpg]

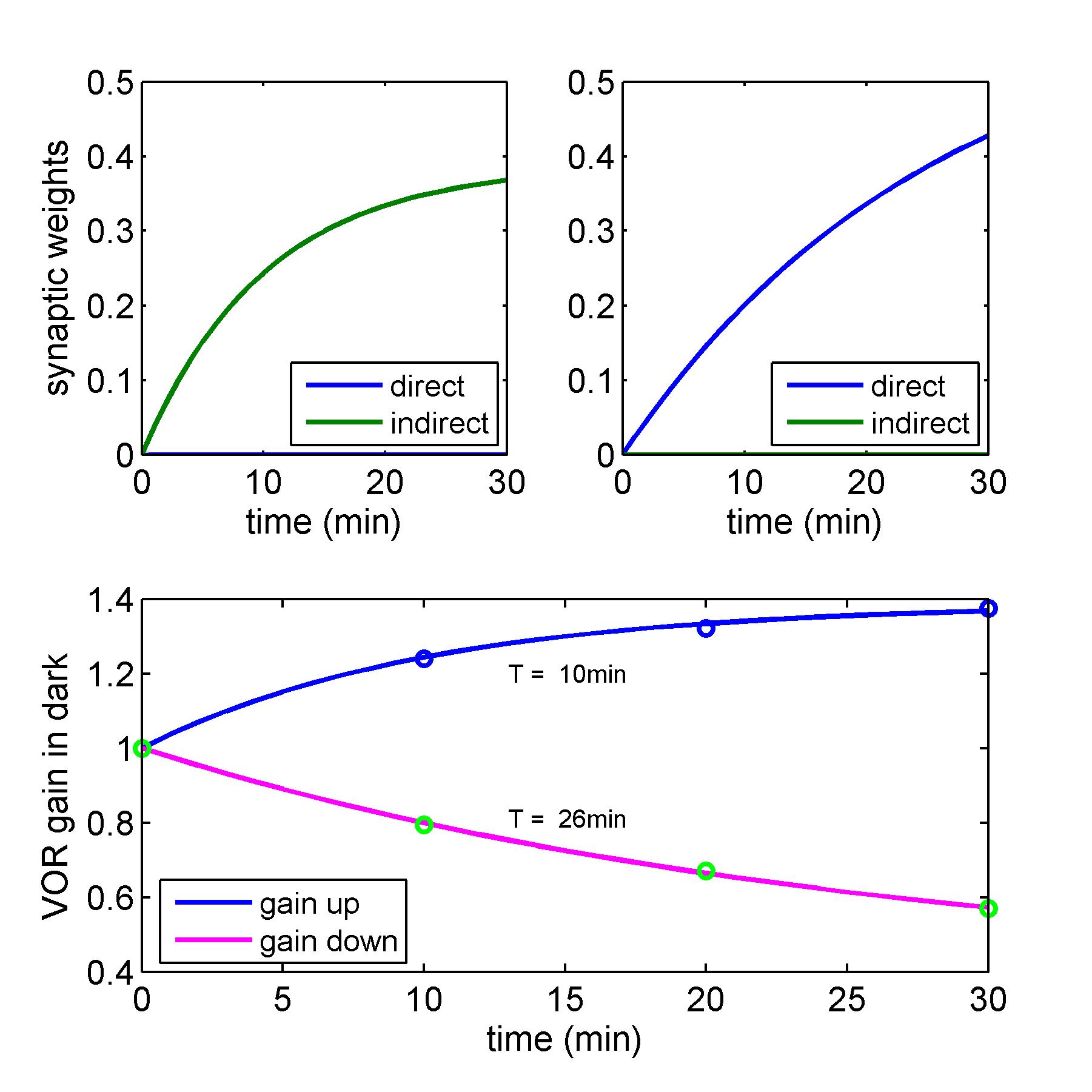

Supplement: Supplementary file 1 [file pcbi.349e7f3a-c9ce-4de2-b369-254ae8ab76cd.s001.zip › code/fig5/fig5.jpg]

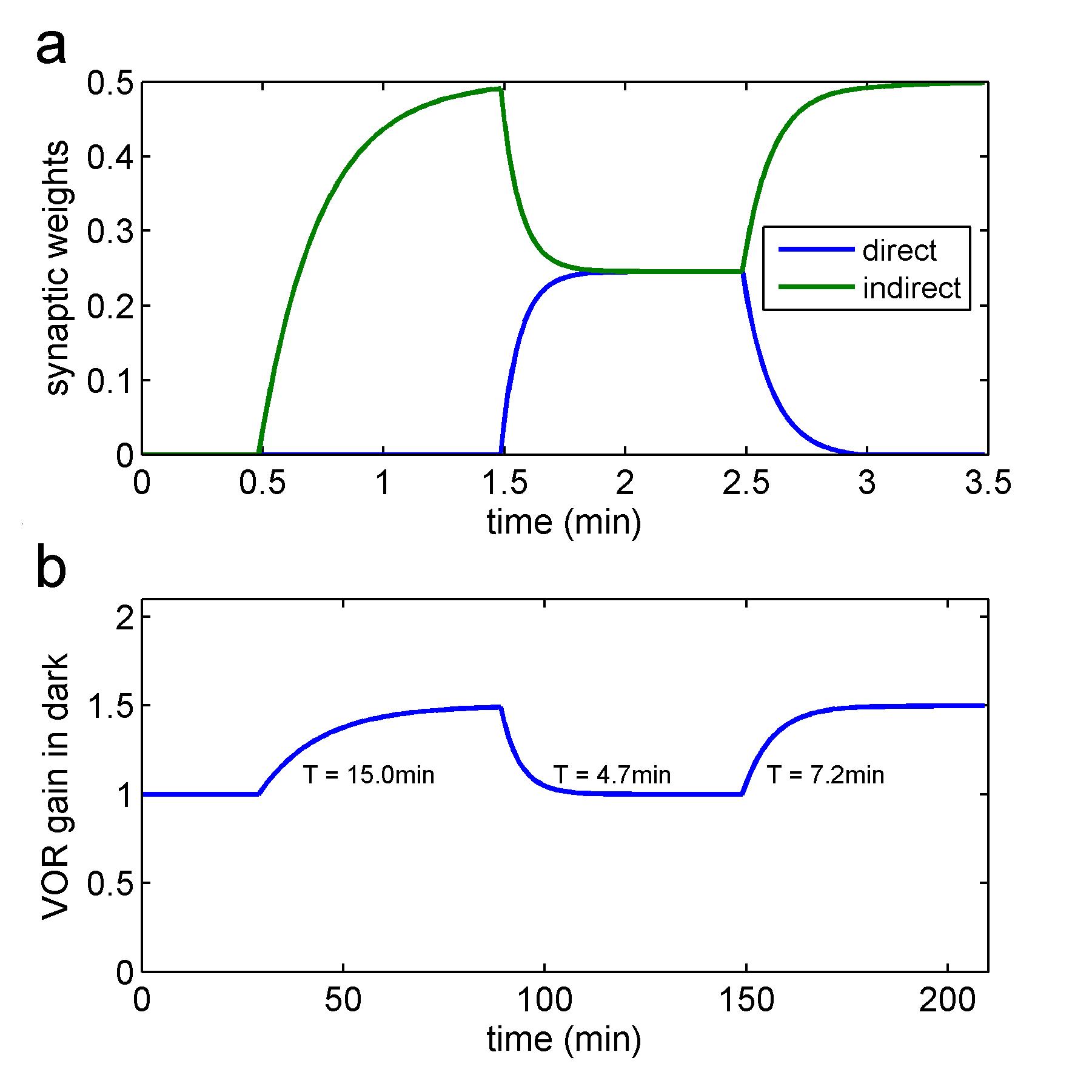

Supplement: Supplementary file 1 [file pcbi.349e7f3a-c9ce-4de2-b369-254ae8ab76cd.s001.zip › code/fig6/fig6.jpg]
